# An exploration of alginate oligosaccharides modulating intestinal inflammatory networks *via* gut microbiota

**DOI:** 10.3389/fmicb.2023.1072151

**Published:** 2023-01-26

**Authors:** Zhikai Zhang, Xuejiang Wang, Feng Li

**Affiliations:** Wuzhoufeng Agricultural Science and Technology Co., Ltd., Yantai, China

**Keywords:** alginate oligosaccharides, gut microbiota, inflammation network, T helper cells, cytokines, probiotics

## Abstract

Alginate oligosaccharides (AOS) can be obtained by acidolysis and enzymatic hydrolysis. The products obtained by different methods have different structures and physiological functions. AOS have received increasing interest because of their many health-promoting properties. AOS have been reported to exert protective roles for intestinal homeostasis by modulating gut microbiota, which is closely associated with intestinal inflammation, gut barrier strength, bacterial infection, tissue injury, and biological activities. However, the roles of AOS in intestinal inflammation network remain not well understood. A review of published reports may help us to establish the linkage that AOS may improve intestinal inflammation network by affecting T helper type 1 (Th1) Th2, Th9, Th17, Th22 and regulatory T (Treg) cells, and their secreted cytokines [the hub genes of protein–protein interaction networks include interleukin-1 beta (IL-1β), IL-2, IL-4, IL-6, IL-10 and tumor necrosis factor alpha (TNF-α)] *via* the regulation of probiotics. The potential functional roles of molecular mechanisms are explored in this study. However, the exact mechanism for the direct interaction between AOS and probiotics or pathogenic bacteria is not yet fully understood. AOS receptors may be located on the plasma membrane of gut microbiota and will be a key solution to address such an important issue. The present paper provides a better understanding of the protecting functions of AOS on intestinal inflammation and immunity.

## Introduction

The human gut contains over 100 trillion microorganisms, including gram-negative and gram-positive bacteria, archaea, bacteriophages, fungi, and protozoa (Subramanian et al., 2018; Nishida et al., 2022). Lipopolysaccharides (LPS), which are also known as lipoglycan or endotoxin, are the main components of the outer membrane of Gram-negative bacteria and induce human gut inflammation and obesity development(Du et al., 2022). Endotoxin affects the composition of the intestinal flora, destroy intestinal mucosal barrier, leads to a large increase in the reproduction and translocation of harmful bacteria, increases the serum endotoxin level, and eventually causes endotoxemia (Fuke et al., 2019). The intestinal alterations, including gut barrier dysfunction, dysbiosis, and endotoxemia, will affect intestinal homeostasis (Kühn et al., 2020). Intestinal homeostasis is critical for health, which is dependent on multifaceted interactions between the gut microbiota, the intestinal epithelium and mucosal immune system(Ahlawat et al., 2021). Maintaining the balance of gut microbiota is important to promote intestinal homeostasis(Huang P. et al., 2021).

Intestinal inflammation has been regarded as a serious, worldwide public health issue, and especially inflammatory bowel disease (IBD) is a persistent and worsening inflammatory gut disease (Khan et al., 2019). There are 10 million people globally living with IBD according to the European Federation of Crohn’s and Ulcerative Colitis Associations (EFCCA)(Zhao et al., 2021). Gut microbiota interacts with the host *via* metabolites, such as bile acids, short-chain fatty acids (SCFA) and tryptophan metabolites, which affect host immune development, immune homeostasis, and energy metabolism. Alterations in gut microbiota and their metabolites have been described in much work on IBD (Lavelle and Sokol, 2020). Therefore, gut microbiota imbalance is an important factor in abnormal intestinal inflammation (Lobionda et al., 2019). Fecal microbiota transplantation and probiotic intervention are promising approaches in the prevention of IBD (Dang et al., 2020). Orally administered probiotics can be beneficial to restore dysbiotic microbiota and to prevent obesity or IBD (Lavelle and Sokol, 2020). Nature products present a promising potential to treat IBD by improving the growth of probiotics in gut microbiota (Zhang N. et al., 2021).

Alginate is an active substance derived from the ocean, which is widely present in the cell walls of marine algae and is a polymer compound composed of D-mannuronic acid (M-block) and L-guluronic acid (G-block). Alginate oligosaccharides (AOS) can be obtained from alginate by acidolysis and enzymatic hydrolysis. The products obtained by different methods have different structures and different physiological functions. Alginate can be degraded into AOS with an alginate to water ratio of 1:25 (w/v) and 1.0% formic acid, and the hydrolysate water showed high antioxidant properties (Meillisa et al., 2015). Sulfuric acid hydrolysis is a typical method to prepare AOS, which can promote the growth of *Nannochloropsis oculata* (Park et al., 2011). Alginate is treated with trifluoroacetic acid (TFA) and used in innovative biomedical devices (Hajiali et al., 2015). Alginate lyases play a critical role to produce AOS *via* alginate degradation (Cheng et al., 2020). Enzymatic hydrolysis is the key method to prepare AOS with the specific polymerization degree (DPs) with certain purity and activities (Cao et al., 2021; Ming et al., 2021).

AOS have received increasing attention not only because of its low molecular weight and viscosity but also its good solubility in water, which makes them useful in medicine (Liu J. et al., 2019). AOS possess various applications in food and biomedical industries, and exert multiple health-promoting properties such as anti-inflammatory, anti-microbial, anti-oxidant, and immunomodulation (Wang et al., 2021; Zhang et al., 2023). Meanwhile, many functional oligosaccharides including AOS have been reported as prebiotics to ameliorate ulcerative colitis (UC) *via* the SCFAs produced from the oligosaccharide metabolized by gut microbiota (Liu et al., 2022). AOS have antibacterial infection activities and have been prepared as wound dressings to maintain a physiologically moist environment, and minimize bacterial infections (Aderibigbe and Buyana, 2018). AOS have been reported to exert protective functions for intestinal damage by regulating gut microbiota (Zhang P. et al., 2021), improving immunity (Hu et al., 2021), reducing endotoxemia (Gotteland et al., 2020), and gut inflammation (Zhang P. et al., 2020).

However, intestinal inflammatory disease, such as IBD, is often associated with inflammation networks (Friedrich et al., 2019; Zhang P. et al., 2020). The effects of AOS on the inflammation network remain widely unclear. In this review, AOS are used as the intervention substances, and the effects of AOS on gut homeostasis and inflammation are analyzed by analyzing animal gut microbiota and relevant inflammatory factors. The possible effects of AOS on gut microbiota and inflammation network are explored.

### AOS modulate intestinal homeostasis *via* the regulation of gut microbiota

Gut microbiota consists of pathogenic bacteria and beneficial bacteria, and the balance between them will be critical to maintain gut-healthy status. Here, we tried to explore the effects of AOS on gut probiotics and pathogenic bacteria.

AOS strengthen gut health *via* the metabolites of probiotics.

**Figure 1:**
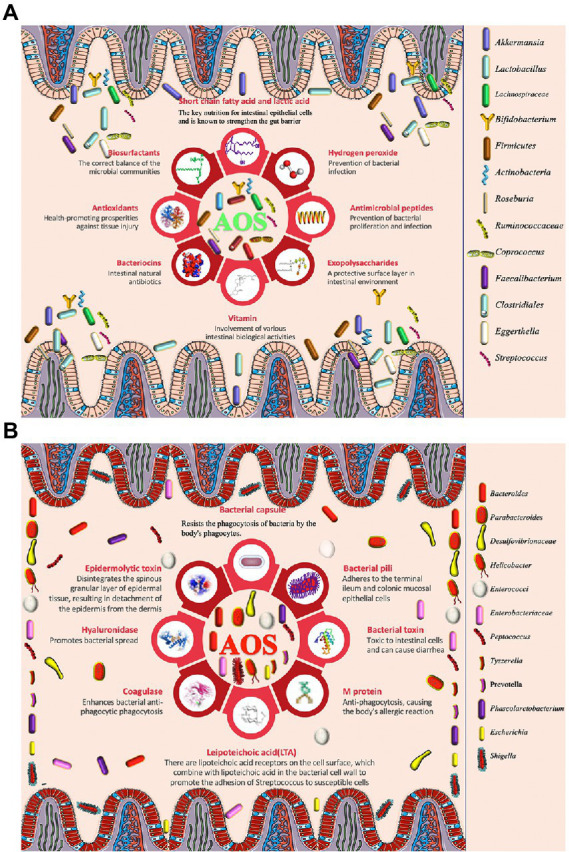
The effects of alginate oligosaccharides (AOS) on intestinal homeostasis *via* modulation of gut microbiota. **(A)** AOS exert protective roles for intestinal homeostasis by increasing the proportion of probiotics. **(B)** AOS exert protective roles for intestinal homeostasis by reducing the proportion of harmful pathogens.

Probiotics produce large amounts of postbiotic metabolites, which play important roles in regulating human health (Pelton, 2020). Vitamin K has been regarded as an underappreciated mediator of gut microbiota community dynamics (Ellis et al., 2021). B vitamins are responsible of crucial microbial bioactivities, metabolism and signaling. Vitamins C, E and B2 are widely reported antioxidants, which affect luminal redox balance (Pham et al., 2021). On the other hand, most gut probiotic, are capable of synthesizing vitamin K and most of B vitamins, including biotin, cobalamin, folates, nicotinic acid, pantothenic acid, pyridoxine, riboflavin, and thiamine (Figure 1A; Gu and Li, 2016). GSH is a major antioxidant and capable of eliminating ROS-caused damage to the most cells, and can be synthesized by *Lactobacillus salivarius* (Yuan et al., 2022). Antimicrobial peptides (AMPs) are a class of small peptides, which play a key role in the innate immune system of gut health (Zong et al., 2020). Some probiotic lactic acid bacteria produce bacteriocins (Figure 1A), a kind of small cationic peptides that kill the pathogen cells *via* pore formation (Tiwari, 2022). Defensins belong to cationic antimicrobial peptides, which prevent bacterial infection (Pero et al., 2019) and are critical elements of innate immunity in gut health (Chung and Raffatellu, 2019; Shulman et al., 2021). Phenyllactic acid, a product of phenylalanine catabolism, is the main bioactive metabolite produced by *Saccharomyces boulardii* (Fu et al., 2022). Phenyl lactic acid improves *Samonella Typhimurium*-induced colitis by modulating regulating the components of gut microbiota, SCFA production and inflammatory activities (Zhou et al., 2021). Volatile organic compounds (VOCs) are well-known biomarkers of gastrointestinal diseases and nutritional situation (Rondanelli et al., 2019). *Bacillus amyloliquefaciens* and some yeasts synthesize high level of VOCs (Ngo et al., 2020; Shruthi et al., 2022). AOS has been report to affect the most related lactic acid bacteria (Zhuge et al., 2020; Le et al., 2021; Yudiati et al., 2021), yeasts (Chávez-Falcón et al., 2022), and other probiotics (Guleria et al., 2016) under simulated gastrointestinal conditions.

*Akkermansia muciniphila* is considered to be favorable intestinal probiotics by improving gut metabolic activities and immune capacity (Zhang et al., 2019; Xu et al., 2020). Both probiotics *Lactobacillus gasseri* and *Lactobacillus reuteri* strains have clinically proven to have health-promoting effects by improving lipid metabolism and inflammation (De Gregorio et al., 2020; Wang et al., 2020). AOS intervention promotes the growth of *A. muciniphila* (Table 1), *L. reuteri* (Table 2), and *L. gasseri* (Table 2), which play important roles in the improvement of lipid metabolism (Wang et al., 2020). Meanwhile, AOS diet increases concentrations of the metabolites, such as SCFA *via* gut microbiota (Tables 1, 2; Mizuno et al., 2020), including acetic acid, propionic acid, and butyric acid (Wang et al., 2020). SCFAs are the main metabolites of gut microbiota, and closely associated with intestinal barrier integrity. SCFAs exert protective functions for intestinal homeostasis by strengthening gut barrier (Figure 1A; De Gregorio et al., 2020; Liu P. et al., 2021). The receptors and transporters of SCFA regulate the antibiotic long-term effects on the colonic mucosa and main the insusceptibility to experimental colitis (Holota et al., 2019). SCFAs exert protective effects on intestinal barrier function by inhibiting NOD-, LRR- and pyrin domain-containing protein 3 (NLRP3, expressed predominantly in macrophages) inflammasome and autophagy (Feng et al., 2018).

**Table 1:** The effects of alginate oligosaccharide on gut gram-negative microbiota and cytokines.

Gut microbiota changes	Cytokines or their integrator	Metabolites	Animal model	References
*Bacteroidetes* (+)	p-AMPKα(+), NF-κB p65(−)	SCFAs (+)	Pig	Wan et al. (2020)
*Akkermansia muciniphila* (+)	IL-1β and CD11c(−)	SCFAs (+)	High-fat-diet mice	Wang et al. (2020)
*Bacteroidetes (−)*	TNF-α, COX-2, IL-1β, IL-6, KC *(−)*, IL-10(+)	SCFAs (+)	DSS-induced colitis mice	He et al. (2021)
*Helicobacter* and *Tyzzerella*(−)	TLR-4 and MAPK (−)	D-lactic acid and LPS (−)	Cyclophosphamide-induced mice	Huang et al. (2021a)
*Bacteroidales* (+)	IL-1β and TNF-α(−)	SCFAs (+)	High-fat-diet mice	Zheng et al. (2021)
	IL-10(+)			
	NF-κB p65 IL-1, IL-6, TNF-α, IFN-γ (−)		Porcine intestinal barrier injury	Wan et al. (2021)
	IL-1β, IFN-γ(−)		*Salmonella enteritidis*-infected chickens	Yan et al. (2011)
	IL-10 (+)			
	AMPKα, IL-6 and IFN-γ(−)		High-fat-diet-induced obese zebrafish	Tran et al. (2019)
*Bacteroidales*, *Rikenellaceae*, and *Bacteroidaceae* (+) *Acidaminococcaceae* (−)		SCFAs (+)	Spontaneously hypertensive rats	Han et al. (2021)
Akkermansia muciniphila (+)		SCFAs (+)	High-fat-diet mice	Fu et al. (2021)
*Alloprevotella* (+) and *Helicobacter* (−).		HDL-c(+) TG, TC, BCAAs and AAAs (−)	Streptozotocin (STZ)-induced type 2 diabetes mice	Liu J. et al. (2021)
*Mucispirillum* (+)			High-salt-induced liver injury mice	Zhang Z. et al. (2022)
*Prevotella* (+)	IL-10 and TLR-3(+)		Liver injury rats	Zhuge et al. (2020b)
Bacteroidales (+)			High-fat-induced obese mice	Zhang P. et al. (2022)
Bacteroidales (+) Mucispirillum (−)		DHA, EPA and PUFAs(+)	High-fat-induced obese mice	Hao et al. (2022a)
Bacteroides	CD11b^+^F4/80^+^CX_3_CR1^low^Ly6C^+^ cells (−)		High-fat-diet-induced mice	Ejima et al. (2021)
Bacteroidetes and *Akkermansia* (+)	TNF-α, IL-1β, IL-6, and PAI-1(−)		ICR mouse	Takei et al. (2020)
Bacteroides and Parabacteroides (−)	IL-10(+)		High-fat-induced mice	Li et al. (2020)

dextran sulfate sodium (DSS); fumonisin B1(FB1); branched-chain amino acids (BCAAs); aromatic amino acids (AAAs); fatty acid esters of hydroxy fatty acids (FAHFAs); n-3 polyunsaturated fatty acid (PUFA) docosahexaenoic acid (DHA); eicosapentaenoic acid (EPA); n-3 polyunsaturated fatty acids (PUFAs); Institute of Cancer Research (ICR); interleukin-1-beta (IL-1β); interleukin-6 (IL-6); interleukin-10 (IL-10); tumor necrosis factor-alpha (TNF-α); and plasminogen activator inhibitor-1 (PAI-1); lipopolysaccharide (LPS); Toll-like receptor 4 (TLR4); Mitogen-activated protein kinase (MAPK). (+) stands for increase or upregulation and (−) stands for reduction or down-regulation.

**Table 2:** The effects of alginate oligosaccharide on gut gram-positive microbiota and cytokines.

Gut microbiota changes	Cytokines or their integrator	Metabolites	Animal model	References
*Firmicutes* (+)	p-AMPKα(+), NF-κB p65(−)	SCFAs (+)	Pig	Wan et al. (2020)
*Lactobacillus reuteri*, *Lactobacillus gasseri* (+)	IL-1β and CD11c(−)	SCFAs (+)	High-fat-diet mice	Wang et al. (2020)
*Firmicutes* and *Actinobacteria*(+)	TNF-α, COX-2, IL-1β, IL-6, and KC *(−)*, IL-10(+)	SCFAs (+)	DSS-induced colitis mice	He et al. (2021)
*Lactobacillus*, *Roseburia*, and Lachnospiraceae (+)*, Peptococcus* (−)	TLR-4 and MAPK (−)	Serum d-lactic acid and LPS (−)	Cyclophosphamide-induced mice	Huang et al. (2021a)
Clostridiales(−)	IL-1β and TNF-α(−) IL-10(+)	SCFAs (+)	High-fat-diet mice	Zheng et al. (2021)
*Roseburia*, *Bifidobacterium*, and *Turicibacter.*		SCFAs (+)	FB1-induced intestine injury mice	Li et al. (2022)
*Lactobacillus*, *Bacteroides*, *Akkermansia*, *Weissella*, and *Enterorhabdus* (+) *Turicibacter* (−).		HDL-c(+) TG, TC, BCAAs and AAAs (−)	High-fat/STZ-induced type 2 diabetes mice	Liu J. et al. (2021)
*Lactobacillus* (+)	IL-6(−)		Mice	Yu et al. (2022)
*Lactobacillus johnsonii* and *Lactobacillus reuteri* (+).	IL-1β, IFN-γ(−)	FAHFAs(+)	Cisplatin-induced kidney injury mice	Zhang et al. (2022b)
*Lactobacillus* (+).		DHA, EPA (+)	Type 1 diabetic mice	Hao et al., (2022b)
*Lactobacillus, Bifidobacterium, Faecalibaculum*(+)			High-salt-induced liver injury mice	Zhang Z. et al. (2022)
*Bifidobacterium* and *Lactobacillus*(+)	Type 2 macrophage, IL-6, IL-1*β*, TNF-*α*(−), TGF-β(+)		DSS-induced colitis mice	Liu H. et al. (2021)
*Ruminococcaceae, Coprococcus, Roseburia*, *Faecalibacterium*		SCFAs (+)	Pigs	Han et al. (2019)
*Ruminiclostridium*, *Dorea Ruminococcaceae* (−) *Ruminococcaceae*, *Eubacterium* (+)	IL-10 and TLR-3(+)		Acute liver injury rats	Zhuge et al. (2020b)
*Clostridiales* and *Lactobacillales* (−)			High-fat-induced obese mice	Zhang P. et al. (2022)
Firmicutes (−)			*ICR mouse*	Takei et al. (2020)
*Lactobacillus* (+)	TNF-α, IL-1β, IL-6, and PAI-1(−), IL-10(+)		High-fat-induced mice	Li et al. (2020)

dextran sulfate sodium (DSS); fumonisin B1(FB1); branched-chain amino acids (BCAAs); aromatic amino acids (AAAs); fatty acid esters of hydroxy fatty acids (FAHFAs); n-3 polyunsaturated fatty acid (PUFA) docosahexaenoic acid (DHA); eicosapentaenoic acid (EPA); n-3 polyunsaturated fatty acids (PUFAs); Institute of Cancer Research (*ICR);* interleukin-1-beta (IL-1β); interleukin-6 (IL-6); interleukin-10 (IL-10); tumor necrosis factor-alpha (TNF-α); and plasminogen activator inhibitor-1 (PAI-1); lipopolysaccharide (LPS); Toll-like receptor 4 (TLR4); Mitogen-activated protein kinase (MAPK). (+) stands for increase or upregulation and (−) stands for reduction or down-regulation.

SCFA-producing bacteria include *Lactobacillus* (Kusumo et al., 2019)*, Bifidobacterium* (Fang et al., 2021), *Clostridiales* (Gargari et al., 2018), and *Lachnospiraceae* species (Vacca et al., 2020). Bacteroides have been regarded as the predominant genus in the gastrointestinal tract and are associated with a higher concentration of beneficial SCFA (Fernandez-Julia et al., 2021). AOS may stimulate the growth of *Bacteroides* and *Lachnospiraceae* species (Cherry, 2020). Alginate microcapsules are reported to improve the bioactivities of *Bifidobacterium* species (Zhang Z. et al., 2021). Other work also shows AOS treatment increases the probiotic species *Lactobacillus* (Table 2) and *Akkermansia* species (Table 1), and reduces pathogenic species *Bacteroides* and *Parabacteroides* (Figure 1A; Table 1; Li et al., 2020). Moreover, the correlation analysis shows AOS improve gut homeostasis by increasing SCFAs production *via* the probiotics *Roseburia*, *Bifidobacterium* (Table 2), and *Akkermansia* (Table 1) in the model with fumonisin B1-induced intestinal damage (Figure 1A; Li et al., 2022). Other work indicates that AOS treatment maintains mucosal barrier function and inhibits immune injury by increasing *Firmicutes* and *Actinobacteria* and reducing *Bacteroidetes* species (He et al., 2021). AOS increase the proportions of SCFA probiotic producers by increasing the abundance of *Ruminococcaceae, Coprococcus, Roseburia*, and *Faecalibacterium* (Figure 1A; Table 2; Han et al., 2021). AOS has been found to reduce *Salmonella* colonization and promote the improvement of intestinal barrier in broiler chickens (Table 1; Yan et al., 2011). AOS ameliorate high-salt-induced intestinal injury by increasing barrier and absorption functions by increasing the abundance of *Lactobacillus, Bifidobacterium, Faecalibaculum* (Table 2) and *Mucispirillum* (Table 1; Zhang Z. et al., 2022). AOS Administration significantly upregulate the levels of IL-10 and TLR-3, and gut barrier biomarkers claudin-1 and mucin 2 (MUC2; Zhuge et al., 2020). MUC2 is a key secretory protein observed in the human intestinal system (Liu et al., 2020).

AOS improve antibacterial infection of the intestine.

Figure 1A shows AOS may improve gut microbiota by inducing probiotics producing biosurfactants, SCFA, hydrogen peroxide, antimicrobial peptides, expolysaccharides, vitamin and antioxidants so on. Biosurfactants are active compounds that are produced from cell surface and the most biosurfactants obtained from a large number of lactic acid bacteria. Probiotic biosurfactants exert beneficial biological activity on the gut microbiome and against pathogen infection *via* an immense antimicrobial, anti-adhesive, and antibiofilm potential (Figure 1A; Patel et al., 2021; Satria, 2022). Exopolysaccharides are the long-chain polymers of carbohydrates and can produce a protective surface layer in intestinal environment for gut microbiota (Figure 1A). Exopolysaccharide from *L. rhamnosus* controls dextran sulfate sodium (DSS) -induced colitis in mice by improving gut microbiota (Wan et al., 2022). Probiotics also produce hydrogen peroxide (Figure 1A), which plays a critical role in the treatment of *H pylori* infection (Alipour and Mofarrah, 2022; Nabavi-Rad et al., 2022).

*Staphylococcus aureus* can cause a wide variety of infections from skin to life-threating infections (Scolari et al., 2020). It has been widely reported that *S. aureus* infections are associated with intestinal symptoms, and its influence may be related to lipid raft-associated trafficking of sucrase–isomaltase and thereby may trigger secondary functional gastrointestinal diseases (Mergani et al., 2021). The film which is made of AOS shows the antimicrobial activity against two common pathogenic bacteria *S. aureus* and *E. coli* and two pathogenic fungi *Aspergillus niger* and *Penicillium digitatum* (Aloui et al., 2021). AOS films are also found to prevent *S. aureus* and methicillin-resistant *S. epidermidis* infections by inducing very high antibacterial activity against these life-threatening pathogens (Martí et al., 2019). AOS exert protective functions against enterotoxigenic *E. coli*-induced animal intestinal barrier injury and its infection (Wan et al., 2021).

Diarrhea is the main symptom of intestinal bacterial infection. AOS have been found to prevent diarrhea by regulating the abundance of *Alloprevotella, Bacteroides*, *Parabacteroides* and *Rikenellaceae* (Figure 1A; Yao et al., 2021). The probiotic mixtures with *L. casei, L. bulgaricus*, and *Streptococcus thermophiles* can prevent diarrhea in the elderly (Figure 1A; Mallina et al., 2018). There is a synergic effect between AOS and probiotics (*L. bulgaricus* and *S. thermophilus*; Yudiati et al., 2021).

Figure 1B shows that AOS also reduce the abundance of some pathogenic bacteria, which include *Escherichia, Shigella*, and *Peptoniphilus* species so on (Figure 1B; Han et al., 2021). These pathogens threaten gut health by producing bacterial toxin (Asadpoor et al., 2021a), hyaluronidase (Tomlin and Piccinini, 2018), lipoteichoic acid (Szentirmai et al., 2021), M protein(Kolesinski et al., 2022), and other structures (bacterial capsule and pili) to help them stay in gut tracts (Gupta et al., 2019). Some pathogens are closely associated with intestinal infection and diarrhea (Figure 1B; Peh et al., 2022). Bacterial toxin produced by the pathogenic species may be the main reason for causing diarrhea (Figure 1B; Dubreuil, 2019).

AOS improve antioxidant properties of the intestine.

AOS increase the abundance of *Clostridium orbiscindens, Ruminococcus gnavus, Eggerthella lenta, Clostridium* spp. and *Clostridiales* species in intestine by improving their fermentation levels (Figure 1A; An et al., 2013). *Clostridium butyricum* increases intestinal antioxidant properties and resistance to adverse stress (Duan et al., 2019). Gut *Clostridia* species display antioxidant activities by producing the antioxidants glutathione, ascorbic acid and uric acid (Million et al., 2020). The probiotics *L. casei, L. bulgaricus*, and *Streptococcus thermophiles* also show antioxidant functions in the elderly (Figure 1A; Mallina et al., 2018; Shori et al., 2022). AOS significantly repair FB1-induced intestinal damage, inflammation, and oxidative stress (including T-SOD and MDA) by increasing the probiotic abundance, such as *Roseburia, Bifidobacterium*, and *Akkermansia*, and SCFAs production (Li et al., 2022). SCFAs, such as acetic, propionic, and butyric acids, can exert health-promoting properties by increasing antioxidant activities. Acetic acid promotes the enzymatic antioxidant ability and stimulates antioxidant responses (Gurdo et al., 2018). Propionic exerts anti-inflammatory and antioxidant properties in addition to its antimycobacterial activity. It has therapeutic potential for the treatment of the patient populations driven by excessive inflammation and tissue damage (Negatu et al., 2020). Sodium butyrate is found to increase the oxidative status by activating of Nrf2-dependent signaling (Ma et al., 2018). AOS improve oxidative stress in kidney-damaged model by increasing the levels of SOD and CAT, and reducing the levels of MDA by increasing the abundance of *L. johnsonii* and *L. reuteri*.

AOS increase anti-inflammatory properties of the intestine by improving gut microbiota.

AOS also reduce the abundance of pathogenic bacteria including *Escherichia*, *Shigella*, and *Peptoniphilus* species (Han et al., 2021), which are closely associated with intestinal inflammation (Peh et al., 2022). AOS protect against intestinal injury by decreasing the abundance of *Enterobacteriaceae, Enterococci* (Wang et al., 2006), *Bacteroidetes* (He et al., 2021), *Desulfovibrionaceae*, *Helicobacter, Peptococcus*, and *Tyzzerella* in the intestine (Figure 1B; Huang J. et al., 2021). The abundance of *Bacteroides* is negatively associated with the amounts of inflammatory monocytes and positively linked with the levels of the metabolites in intestine (Ejima et al., 2021). Most of these species have capsules to resist bacterial phagocytosis (Figure 1B) and can induce intestinal inflammation and affect intestine permeability (Zhang Y. et al., 2020). AMP-activated protein kinase (AMPK) and NF-κB p65 are critical integrators of cytokine signals and have been observed to be reduced after AOS intervention (Tran et al., 2019; Wan et al., 2020, 2021; Huang J. et al., 2021). AOS can improve obesity-related metabolic abnormalities and inflammation. AOS intervention reverses the gut dysbiosis by increasing the relative abundance of *Lactobacillus* (Table 2) and *Akkermansia* species (Table 1) and decreasing the abundance of *Bacteroides* and *Parabacteroides* species (Table 1; Li et al., 2020b). AOS supplementary diet reduces the levels of inflammatory cytokines IL-1β and CD11c. AOS supplement ameliorates the inflammatory responses in a DSS-induced colitis model by reducing the levels of TNF-α, COX-2, IL-1β, IL-6, and increasing IL-10 level *via* the regulation of the abundance of *Firmicutes, Actinobacteria*, and *Bacteroidetes* (He et al., 2021). AOS reduce gut inflammation by decreasing D-Lactic acid and LPS levels, and TLR-4 *and* MAPK expression. Furthermore, AOS also considerably improves the abundance of *Lactobacillus*, *Roseburia*, and *Lachnospiraceae*) and reduce the abundance of *Helicobacter*, *Peptococcus*, and *Tyzzerella* (Huang J. et al., 2021).

### AOS regulate intestinal inflammation network *via* changes of metabolites of gut microbiota

**Figure 2:**
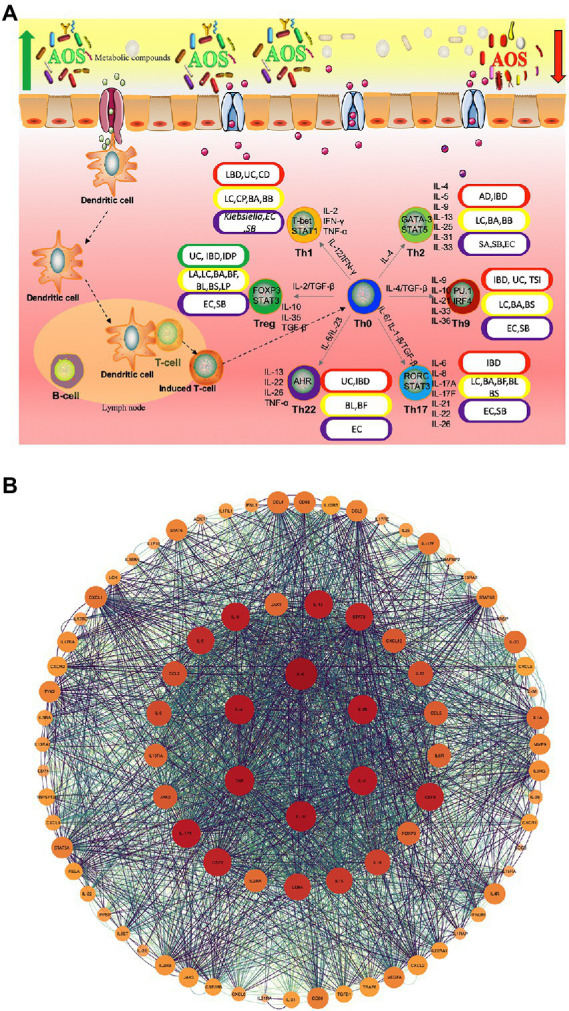
The roles of AOS in the inflammatory immunology process of intestine possible by increasing the beneficial metabolites of probiotics. **(A)** AOS may improve intestinal inflammation network by affecting T helper cells and regulatory T (Treg) cells and their secreted cytokines. Red, yellow, purple and green capsules stand for the occurrence of intestinal diseases, probiotics, pathogens and reduction in intestinal diseases because of different Th-type cell responses. **(B)** protein–protein interaction (PPI) networks are constructed *via* webservice String (Szklarczyk et al., 2021) and visualized *via* Cytoscape software (Doncheva et al., 2018). IBD, Inflammatory bowel disease; UC, ulcerative colitis; CD, Crohn ‘s disease; AD, allergic diarrhea; TSI, Trichinella spiralis infection; IDP, intestinal dysplastic progression; BA, *Bifidobacterium adolescentis*; BB, (*B*)*. breve*; BS, *B. spp*; BL, *B. longum* DJO10A; BF, *Bacteroides fragilis*; LC, *Lactobacillus casei*; LC, (*L*)*. casei* CRL431; LP, *L. paracasei* CNCMI-1518; LA, *L. acidophilus*; SA, *Staphylococcal aureus*; EC, *E. coli*; SB, *Saccharomyces boulardii*.

Although anti-inflammatory properties of AOS have been widely reported, the roles of AOS in intestinal inflammation networks remain widely unclear. Figure 2A shows AOS may improve intestinal inflammation cells [Th1, Th2, Th9, Th17, Th22 and regulatory T (Treg) cells] and their secreted inflammatory cytokines *via* SCFA, butyrate and essential metabolites produced from probiotics. These factors are closely associated with various gut inflammatory diseases or gut health. T Helper (Th) Cells (T helper type 1 (Th1; IFN-γ, IL-2, and TNF-β; Wang J. et al., 2018; Pradhan et al., 2019), Th2 (IL-4, IL-5, IL-9, IL-10, IL-13, IL-25, IL-31, and IL-33; Pradhan et al., 2019), Th9 (IL-9, IL-10, IL-21, IL-33, and IL-36; Hoeppli et al., 2019), Th17 (IL-6, IL-8, IL-17A, IL-17F, IL-21, IL-22, and IL-26; Kulkarni et al., 2018; Wang J. et al., 2018; Alrafas et al., 2019), Th22 (IL-13, IL-22, IL-26 and TNF-α; Sanaii et al., 2019; Shohan et al., 2020) and regulatory T (Treg) cells (IL-10, TGF-β and IL-35; Kulkarni et al., 2018; Alrafas et al., 2019) are involved with gut inflammation (Figure 2A). The effects of AOS on these cells or cytokines are explored. Th cell responses may be affected by the metabolites of probiotics during the prevention of gut inflammation (Figure 2A; Liu X.-J. et al., 2019; Di Gangi et al., 2020). Figure 2B shows the protein–protein interaction (PPI) networks of main inflammatory cytokines, which are also closely associated with gut inflammation and health and may be affected by AOS treatment. Among their secreted cytokines, Cytoscape analysis shows the hub genes of protein–protein interaction networks include IL-10, IL-6, IL-4, IL-2, IL-1β, and TNF-α (Figure 2B), which are closely associated with gut inflammation and health. AOS treatment enhances IL-10 secretion by affecting gut microbiota when compared with LPS treated animal models (Zhang et al., 2022a). AOS intervention decreases the levels of IL-1β, IL-6, and TNF-α, and increases the levels of IL-10 (Zhang et al., 2022a). AOS treatment affects the levels of Th1 cytokines (IL-2 and IFN-γ), and Th2 cytokines (IL-4 and IL-6; Wang W. et al., 2018). IL-10-deficiency will induce colitis in an animal model (Kang et al., 2018) while deletion of IL-6 can exacerbate colitis by inducing systemic inflammation (Ye et al., 2020). IL-1β plays an important role in the pathogenesis of IBD (Mao et al., 2018). IL-2 induces colitis by activating STAT5, which is required for optimal IL-22 production (Bauché et al., 2020). Circulating pro-inflammatory cytokine Il-4 is found to be increased in an IBD model (Zhou et al., 2019). TNF-α-producing CD4+ effector memory T cells stimulate intestinal development and regulate inflammatory responses (Schreurs et al., 2019). Anti-TNF-α therapy inhibits proinflammatory activities of mucosal neutrophils in IBD (Zhang et al., 2018).

AOS can affect many different types of T helper cells and regulate immune-induced diseases. AOS and their derivatives have been found to reduce allergic responses by altering Th1/Th2 balance forward to Th1 cells, inhibiting IgE production, and maintaining the amounts of mast cells (Vo et al., 2015). Oral administration of β-d-mannuronic acid (the main component of AOS) down-regulates the levels of Th17 cell, IL-17 and IL-6 in patients with ankylosing spondylitis (Figure 2; Fattahi et al., 2018). AOS can significantly increase the immunosuppressive activity of Treg cells (CHEN and YAO, 2019).

AOS can increase the abundance of most *Lactobacillus* species in intestine. *L. casei* CRL431 and *L. paracasei* CNCMI-1518 have been found to show protective function against *Salmonella typhimurium*, which can cause Th1-type cell-mediated immunity by increasing IFN-γ/IL-4 ratio (Figure 2; Lemme-Dumit et al., 2021). The over-expression of IL-12p40 contributes to Th2-type inflammatory responses in the large intestine of mice with allergic diarrhea (Hino et al., 2004). IBD, including ulcerative colitis (UC) and Crohn’s disease (CD), are related to the imbalances of gut microbiota. *B. adolescentis* regularly treatment may improve the therapeutic effects for IBD by stimulating protective Treg/Th2 response and gut microbiota remodeling (Fan et al., 2021). Th9 cells also promote IBD risk by increasing IL-9 levels and *Bifidobacterium* species may be related to the prevention of IBD (Vyas and Goswami, 2018; Jakubczyk et al., 2020). The pro-inflammatory responses induced by Th1, Th2, and Th17 is also associated with IBD pathogenesis. IL-25, an IL-17 regulate Th2- and Th9-type immune responses, and IL-25 is important cytokine against *T*. *spiralis* infection (Angkasekwinai et al., 2017). AOS may also affect Th9- and Th17-type inflammatory responses by increasing the abundance of *Bifidobacterium* species (Figure 2; Li et al., 2022).

Th22 cells are closely associated with the severity of IBD, and may be involved in the inflammatory process of IBD (Xia and Li, 2019). Th2/Th17/Th22 responses are common to *E. coli*-derived vesicles but specific differences are found in Th1 and Treg cell responses (Diaz-Garrido et al., 2019). The balance of Th17/Treg cells plays an important role in the prevention of IBD progression and development (Yan et al., 2020). *Lactobacillus acidophilus* has been found to inhibit IBD development by regulating the balance between Th17 and Treg cells (Park et al., 2018). *Bifidobacterium longum* and *L. plantarum* alleviate allergic rhinitis in mice by revering Th2/Treg balance (Kim et al., 2019). AOS extend the viability of *Lactobacillus* species (Tiani et al., 2018), increase the abundance of *Bifidobacterium* species (Li et al., 2022), reduce the abundance of *E. coli* (Han et al., 2021), and may also affect Th2/Th17/Th22/Treg cell response (Figure 2). All the results suggest that there may exist a linkage between AOS and Th cells or their cytokines *via* the regulation of gut microbiota.

AOS show protective roles for intestinal homeostasis by increasing the abundance of probiotics (Table 1), which are linked with reducing intestinal inflammation (Zhang T. et al., 2021), improved gut barrier (Ottman et al., 2017), reduced bacterial infection (Peng et al., 2020), against tissue injury (Si et al., 2022), the balance of gut microbiota (Aabed et al., 2019), and protective surface layers (Meng et al., 2021; Figure 1A). However, the most other functions are not deeply explored in the present study. For instance, AOS show anti-adhesive properties by hampering pathogenic bacteria *E. coli* (Asadpoor et al., 2021b). Bacterial adhesion pili is also associated with pathogenic adhesion in intestine (Figure 1B; Shori et al., 2022) but the possible association with AOS intervention has been seldom reported. There are many great challenges to elucidate the mechanism for the effects of AOS on intestinal homeostasis and inflammation networks. The exact mechanism for the direct interaction between AOS and gut probiotics or pathogenic bacteria has not been established yet. There may be some different receptors between probiotic and pathogenic bacteria, which show different susceptibilities with AOS. Further investigation in IPEC-J2 cells found that AOS acts its function through mannose receptor signaling pathway (Zhao et al., 2020). The polysaccharide utilization loci have been found in Bacteroides species, which may include secreted glycosidases, a complement of cell surface glycan-binding proteins, oligosaccharide receptor or transporters, and a series of metabolic enzymes (Zafar and Saier, 2021). Such a receptor is expected to be discovered in some gut probiotics or gut pathogens to address the important issue.

## Conclusion

AOS have caught increasing attention recently because of their antioxidant and anti-inflammatory properties. AOS also exert their numerous functions for gut homeostasis by reducing intestinal inflammation, bacterial infection, and tissue injury, increasing biological responses and improving gut barrier. AOS may affect intestinal inflammation network by regulating the levels of Th1, Th2, Th9, Th17, Th22, and regulatory T (Treg) cells, and their secreted cytokines *via* the increase in the proportion of probiotics. However, the mechanism for the direct interaction between AOS and probiotics or pathogenic bacteria remains unclear. Possible existence of some AOS receptors on the outer membrane of gut microbiota may provide a key clue to explore the mechanism. Much work needs to be done to address such an important issue in the future.

## Author contributions

ZZ and XW were involved in the initial conceptualization of this manuscript. ZZ and FL led the literature review, writing of the first draft, and involved in the visualization of concepts. XW provided revisions and additional conceptual input into the manuscript. All authors contributed to the article and approved the submitted version.

## Funding

This study received funding from Wuzhoufeng Agricultural Science and Technology Co., Ltd. The funder was not involved in the study design, collection, analysis, interpretation of data, the writing of this article, or the decision to submit it for publication.

## Conflicts of interest

Authors ZZ, XW, and FL were employed by Wuzhoufeng Agricultural Science & Technology Co., Ltd.

## Publisher’s note

All claims expressed in this article are solely those of the authors and do not necessarily represent those of their affiliated organizations, or those of the publisher, the editors and the reviewers. Any product that may be evaluated in this article, or claim that may be made by its manufacturer, is not guaranteed or endorsed by the publisher.
